# Longitudinal shortening remains the principal component of left ventricular pumping in patients with chronic myocardial infarction even when the absolute atrioventricular plane displacement is decreased

**DOI:** 10.1186/s12872-017-0641-z

**Published:** 2017-07-28

**Authors:** Daniel Asgeirsson, Erik Hedström, Jonas Jögi, Ulrika Pahlm, Katarina Steding-Ehrenborg, Henrik Engblom, Håkan Arheden, Marcus Carlsson

**Affiliations:** 1 Department of Clinical Sciences Lund, Clinical Physiology, Lund University, Skane University Hospital, Lund, Sweden; 2 Department of Clinical Sciences Lund, Diagnostic Radiology, Lund University, Skane University Hospital, Lund, Sweden; 3 Department of Health Sciences, Physiotherapy, Lund University, Lund, Sweden

**Keywords:** Regional function, Cardiac output, Heart failure, Mitral annular plane systolic excursion, Late gadolinium enhancement, Cardiac pumping

## Abstract

**Background:**

The majority (60%) of left ventricular (LV) stroke volume (SV) is generated by longitudinal shortening causing apical atrioventricular plane displacement (AVPD) in systole. The remaining SV is caused by radial inward motion of the epicardium both in the septal and the lateral wall. We aimed to determine if these longitudinal, septal and lateral contributions to LVSV are changed in patients with chronic myocardial infarction (MI).

**Methods:**

Patients with a chronic (>3 months) ST-elevation MI in the left anterior descending (LAD, *n* = 20) or right coronary artery (RCA, *n* = 16) and healthy controls (*n* = 20) were examined with cardiovascular magnetic resonance (CMR). AVPD was quantified in long axis cine CMR images and LV volumes and dimensions in short axis cine images.

**Results:**

AVPD was decreased both in patients with LAD-MI (11 ± 1 mm, *p* < 0.001) and RCA-MI (13 ± 1 mm, *p* < 0.05) compared to controls (15 ± 0 mm). However, the longitudinal contribution to SV was unchanged for both LAD-MI (58 ± 3%, *p* = 0.08) and RCA-MI (59 ± 3%, *p* = 0.09) compared to controls (64 ± 2%). The preserved longitudinal contribution despite decreased absolute AVPD was a results of increased epicardial dimensions (*p* < 0.01 for LAD-MI and *p* = 0.06 for RCA-MI). In LAD-MI the septal contribution to LVSV was decreased (5 ± 1%) compared to both controls (10 ± 1%, *p* < 0.01) and patients with RCA-MIs (10 ± 1%, *p* < 0.01). The lateral contribution was increased in LAD-MI patients (44 ± 3%) compared to both RCA-MI (35 ± 2%, *p* < 0.05) and controls (29 ± 2%, *p* < 0.001).

**Conclusion:**

Longitudinal shortening remains the principal component of left ventricular pumping in patients with chronic MI even when the absolute AVPD is decreased.

## Background

Myocardial infarction (MI) leads to decreased left ventricular function due to the necrosis of myocytes and subsequent replacement with fibrous tissue and is the most common etiology to heart failure [1]. Cardiac magnetic resonance (CMR) imaging can quantify both left ventricular function using cine images as well as MI using late gadolinium enhancement (LGE) images. CMR has been used to study the relationship between the size of an MI and the overall left ventricular ejection fraction (LVEF) [2, 3]. The relationship is not straightforward but there is an upper limit of ejection fraction determined by infarct size [4]. Both infarct size and ejection fraction are important prognostic factors after an MI [5]. Myocardial infarction also affects longitudinal ventricular function and this can be evaluated as the atrioventricular plane displacement (AVPD) [6]. A decreased AVPD is associated with worse prognosis after an MI [7]. In echocardiography, measurements of AVPD or mitral annular plane systolic excursion (MAPSE) are used for detection of decreased ventricular function. The interest in longitudinal function has recently increased as global longitudinal strain seem to be a stronger predictor than ejection fraction for mortality [8] and lateral MAPSE from CMR has been shown to be an independent predictive factor for cardiac events [9].

Cardiovascular magnetic resonance imaging has the ability to quantify the amount of stroke volume (SV) generated by longitudinal function by combining the measurements of the AVPD and the short-axis area of the left ventricle encompassed by the AVPD, and express this as a percentage of SV [10, 11]. In healthy controls ~ 60% of the left ventricular (LV) SV is generated by longitudinal function and the rest from radial (circumferential) function seen as the systolic radial inward motion of the epicardium [10–12]. The radial component of LV pumping can be subdivided into the movements of the septum and the remaining anterior, lateral and inferior walls, denoted lateral wall for short. Normal septum movement contributes ~ 8% to LVSV and the lateral wall ~ 30% [13]. The size of an MI affects overall pumping, but it is not known to what extent an MI affects the longitudinal contribution to LVSV and if the size and location of an MI may have impact on this parameter. The rationale of this study was to increase the fundamental physiological understanding of longitudinal left ventricular function in chronic MI patients so that the clinical tools of longitudinal function, e.g. global longitudinal strain and MAPSE can be better understood. Therefore, we aimed to quantify the effect of chronic MI on the longitudinal, septal and lateral contributions to left ventricular pumping.

## Methods

### Study population

This retrospective, cross-sectional study was approved by the Regional ethical committee in Lund and written informed consent was obtained from each subject. Patients with ST-elevation myocardial infarction (STEMI) in the left anterior descending (LAD) territory (*n* = 20, age 59 ± 3 years, 17 males) or the right coronary artery (RCA) territory (*n* = 16, age 57 ± 4 years, 12 males) were studied. Time from MI to CMR examination had to be >3 months for patients to be eligible for inclusion. Exclusion criteria were left bundle branch block, MI within more than one coronary territory, previous cardiac surgery with opening of the pericardium and significant pericardial effusion or valvular disease. Patient characteristics are listed in Table 1. Twenty healthy subjects (age 62 ± 2 years, 12 males) with blood pressure < 140/90, without cardiovascular medications, no previous cardiovascular disease and no ECG abnormalities were used as controls.

**Table 1:** Patient characteristics

Infarction location, *n* (%)	
LAD	20 (56%)
RCA	16 (44%)
Time from MI to CMR (mean ± SE)	5 ± 1 year
Medication *n* (%)	
Acetyl salicylic acid	30 (83%)
Statin	34 (94%)
Betablocker	29 (81%)
ACEi/ARB	30 (83%)
Diuretics	12 (33%)
Warfarin	10 (28%)

### Cardiac magnetic resonance imaging

A 1.5 Tesla CMR scanner was used (Philips Achieva, Best, the Netherlands) to acquire end-expiratory breath held images in the supine position with ECG-triggering. Cine images for LV function were obtained covering the entire heart in the short-axis plane, including both ventricles and atria of the heart using a balanced steady state free precession sequence. Imaging parameters were typically: acquired temporal resolution of 47 ms reconstructed to 30 time phases per cardiac cycle, repetition time 3 ms, echo time 1.4 ms, flip angle 60°, slice thickness 8 mm with no slice gap and SENSE factor of 2. Long-axis images were acquired in the left ventricular two chamber, left ventricular outflow tract and four-chamber planes.

LGE images for infarct quantification were acquired using a 3D inversion-recovery gradient-recalled echo sequence in the short-axis plane 10–20 min after intravenous administration of 0.2 mmol/kg gadolinium-based MR contrast medium. Typical sequence parameters were: repetition time 4 ms, echo time 1.3 ms, flip angle 15°, slice thickness 8 mm, in-plane resolution 1.6 × 1.6 mm with inversion time set to null non-infarcted myocardium.

### Image analysis

All image analysis was done using the software Segment version 1.9 [14]. Delineation of the epicardial and endocardial borders of the LV in end-diastole and end-systole was done to calculate end-diastolic volume (EDV), end-systolic volume (ESV), SV and epicardial short-axis area. Myocardial infarct delineation and quantification of LGE images were done as previously described [15]. In short, the endocardium and epicardium of the LV was delineated and a semi-automatic algorithm was used to quantify infarct size with manual corrections when needed. Infarct size (IS) was measured as a percent of left ventricular mass. The analyzed LGE images were pooled for visualization of extent of infarction for the LAD and RCA populations.

**Fig. 1:**
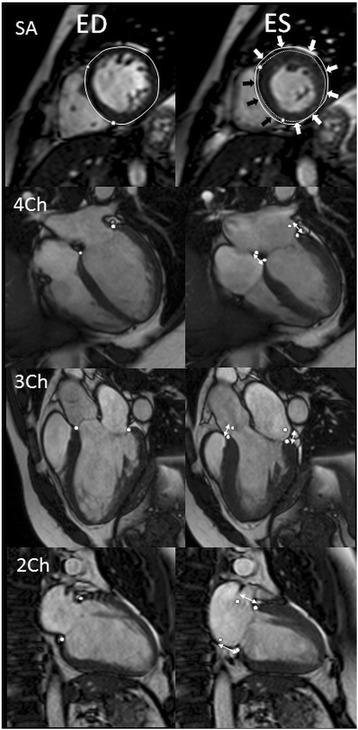
Illustration of the method for quantifying atrioventricular plane displacement (AVPD), septal and lateral contribution to left ventricular stroke volume (LVSV). Short-axis (SA) and four-chamber (4Ch), three-chamber (3Ch) and 2-chamber (2Ch) images are shown in end-diastole (ED) and end-systole (ES). The epicardial border of the SA is shown as a *solid line* in ED and broken line in ES. The ED epicardial contour is transposed on the ES image for comparison. The difference between the epicardial contours in the SA images are defined as the radial pumping and is subdivided into the septal (*black arrows*) and lateral (*white arrows*) contributions as defined by the right ventricular septal insertion points (*marked with circles*). The AVPD was calculated by marking the most basal part of the muscular wall in ED and ES as denoted by the *white circles*. The position of the AV-plane in ED is transposed to the ES image for comparison and the AVPD is marked by the *double-headed arrows*

The longitudinal contribution to LVSV (Fig. 1) was calculated by multiplying the AVPD in systole and the LV epicardial short-axis area from the two largest basal slices, as previously described and validated [11]. The systolic AVPD was measured as the difference from end-diastole to end-systole in the basal part of the LV in two points in each of the three long-axis planes. The AVPD was calculated as the mean of these six measuring points.

The radial contribution to LVSV was defined as the volume difference caused by the inward displacement of the epicardial border of the LV from end-diastole to end-systole and was subdivided into the septal and lateral contributions. The ventricular septum was defined as between the RV insertion points according to the American Heart Association standardization of myocardial segmentation [16]. The LV epicardial contours from short-axis images in end-diastole and end-systole were superimposed in all slices from the base to the apex and generated a volume that represented the volume contributed to SV by the septal movement as previously described [13, 17]. Septal motion towards the LV was considered a positive contribution to the LVSV and septal motion towards the RV was considered a negative contribution to the LVSV. The remaining portion of the radial contribution, i.e. the inward motion of the epicardium from the anterior to the inferior septal insertion point, excluding the septum, was defined as the lateral contribution to SV. Two independent observers measured all AVPD and the short-axis epicardial areas used for calculation of longitudinal contribution.

### Statistical analysis

All statistical analysis was performed using Graphpad Prism v 6.05 (Graphpad Software Inc. La Jolla, USA). Comparisons between the three groups were done with 1-way ANOVA with Tukey’s multiple comparison test. Comparisons between patients for infarct size were done with unpaired t-tests and relationships between continuous variables were tested with Spearman correlation analysis. Results for continuous variables are presented as mean ± standard error of the mean (SE) and results with *P* < 0.05 are considered statistically significant. Internal validations of the measurements are reported as the sum of the longitudinal, septal and lateral contributions to stroke volume, which in theory should be 100%. Inter- and intra-observer variability and the internal validation were calculated as bias ± standard deviation (SD).

## Results

Parameters of LV size and function for the study groups are presented in Table 2. Left ventricular volumes showed a positive correlation with infarct size, EDV = 7.3xIS-15.9, *r* = 0. 75, *p* < 0.001 and ESV = 7.78xIS-1.6, *r* = 0.82, *p* < 0.001. Ejection fraction had a negative correlation with IS, EF = −1.18xIS-0.8, *r* = 0.79, *p* < 0.001. Altogether, patients with MI in the LAD vessel territory had larger volumes and lower EF compared to both patients with RCA-MI and controls (Table 2). The differences between the patient groups were also seen for infarct size with LAD-MI being larger than RCA-MI (*p* < 0.001). RCA-MI patients also had larger EDV and lower EF compared to controls). Atrioventricular plane displacement was decreased in both LAD-MI (11.0 ± 0.7 mm) and RCA-MI (13.0 ± 0.9 mm) compared to controls (15.3 ± 0.4 mm, *p* < 0.001) but did not differ between the patient groups (*p* = 0.08). The epicardial short-axis areas used for calculation of AVPD contribution to SV were increased in infarct patients (*p* < 0.01) but in the subanalysis, LAD-MI had larger epicardial areas compared to controls (*p* < 0.01) while RCA-MI did not reach significance (*p* = 0.06).

**Table 2:** Parameters of left ventricular size and function

	*LAD* *(n = 20)*	*RCA* *(n = 16)*	*Controls* *(n = 20)*
LVEDV, mL	277 ± 3***, ††	202 ± 10**	163 ± 8
LVESV, mL	185 ± 2***, †††	103 ± 7***	66 ± 5
EF, %	37 ± 3***, ††	49 ± 2***	60 ± 1
SV, mL	91 ± 5	98 ± 6	97 ± 4
AVPD, mm	11.0 ± 0.7***	13.0 ± 0.7*	15.4 ± 0.4
SAx Area LV_epi_, cm^2^	49.7 ± 2.7**	44.5 ± 1.7	40.2 ± 1.4
Heart rate, bpm	62 ± 3	66 ± 2	62 ± 2
Infarct size, %	22.3 ± 2.1†††	11.6 ± 1.0	-
Infarct transmurality %	81 ± 2†	75 ± 2	-
Longitudinal contribution LVSV, %	58.5 ± 2.8	58.9 ± 2.7	64.4 ± 1.7
Septal contribution LVSV, %	5.1 ± 1.1**, ††	9.9 ± 0.8	9.5 ± 1.0
Lateral contribution LVSV, %	43.5 ± 3.4†,***	34.7 ± 2.1*	29.2 ± 1.6

*p < 0.05, **p < 0.01 and ***p < 0.001 when comparing patients to controls

†*p* < 0.05, ††*p* < 0.01, and †††*p* < 0.001 when comparing LAD to RCA MI patients

**Fig. 2:**
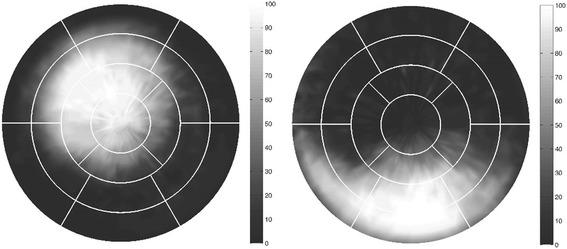
Mean extent of myocardial infarction for the two vessel territories LAD (*left*) and RCA (*right*). Extent is normalized to the population size of each group for comparison between territories, and 0–100% indicate the number of patients having myocardial infarction in a certain area. Overlaid is the standard 17-segment AHA model

Figure 2 shows the mean extent of the MI for all LAD-MI and RCA-MI patients, respectively.

**Fig. 3:**
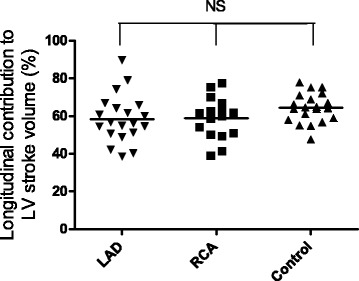
Comparison of the longitudinal contribution to LV stroke volume for patients with MI in the LAD and RCA vessel territories and controls. There was no significant difference between the groups. *Solid line* indicates the mean

**Fig. 4:**
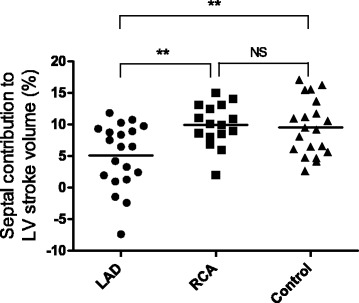
Comparison of the septal contribution to LV stroke volume for patients with MI in the LAD and RCA vessel territories and controls. Septal contribution to SV was significantly lower for the LAD-MI compared to both RCA-MI and controls. There was, however, no significant difference between RCA-MI and controls. Negative values of septal contribution are explained by dyskinesia of the septum, i.e. septal movement toward the RV in systole. *Solid line* indicates the mean. *P < 0.05, **P < 0.01 NS non-significant

**Fig. 5:**
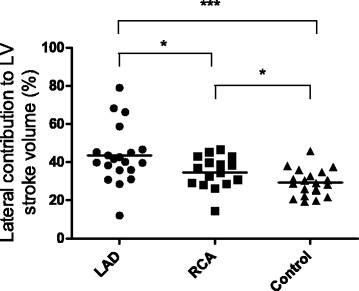
Comparison of the lateral contribution to LV stroke volume for patients with MI in the LAD and RCA vessel territories and controls. Lateral contribution to SV was lower for the RCA-MI compared to LAD-MI. *Solid line* indicates the mean. *P < 0.05, *** *P < *0.001

Longitudinal contribution to LVSV was numerically lower in patients compared to controls but this difference did not reach statistical significance (*p* = 0.08 for LAD and *p* = 0.09 for RCA) (Table 2, Fig. 3). Septal contribution to LVSV was lower in LAD-MI compared to both RCA-infarcts and controls (*p* < 0.01) (Table 2, Fig. 4). Lateral contribution to LVSV was higher for LAD-MI (*p* < 0.001) and RCA-MI (*p* < 0.05) compared to controls (Table 2, Fig. 5). AVPD showed a negative correlation with infarct size in LAD-MI (*r* = −0.59, *p* = 0.006) but not in RCA-MI (*r* = −0.18, *p* = 0.51) patients. However, there was no correlation between infarct size and longitudinal contribution to LVSV in LAD-MI (*r* = −0.114, *p* = 0.63) or RCA-MI (*r* = −0.059, *p* = 0.83) patients. Furthermore, infarct size did not correlate to septal contributions to LVSV in LAD-MI (*r* = 0.08, *p* = 0.74), or in RCA-MI (*r* = −0.45, *p* = 0.08), or lateral contributions to LVSV in LAD-MI (*r* = 0.05, *p* = 0.84) or in RCA-MI (*r* = 0.20, *p* = 0.46).

Internal validation of the measurements by adding the longitudinal, septal and lateral contributions to stroke volume showed 107 ± 16% for LAD-MI, 104 ± 8% for RCA-MI and 103 ± 9% for controls. Interobserver variability for average epicardial areas used for calculation of longitudinal contribution was −0.7±1.7 cm^2^ and for AVPD 0.5±1.0 mm. Intraobserver variability of AVPD in 20 patients analyzed >3 months apart, was 0.3 mm ± 0.7 mm.

## Discussion

This study has shown that longitudinal shortening remains the principal contributor to LV stroke volume in patients with chronic MI even when the absolute AVPD is decreased. This is mainly explained by the LV dilatation after an MI causing a larger short-axis epicardial area. The presence and degree of LV dilatation showed a correlation with infarction size. The three-dimensional intrinsic movement of the myocardium during the cardiac cycle is complex. However, when viewing the LV from the epicardial border, the movement is simpler, comprising of a longitudinal shortening and a radial inward motion [11, 13, 18]. Longitudinal contribution can thus be understood by viewing the LV as a piston pump. The movement of the piston is the AVPD and the volume ejected by longitudinal shortening can be calculated by multiplying the piston movement by the piston area. Thus, if the longitudinal shortening of the ventricle is viewed as a piston, if the “piston” area become enlarged after an MI this can result in an unchanged stroke volume even when the piston movement is decreased.

Of note, the SV and heart rate, were unchanged in MI patients compared to the age-matched controls. Thus, cardiac output was similar in patients and controls. The longitudinal contribution did not differ between infarcts in the LAD or RCA territory. Septal contribution to SV, however, was decreased and lateral contribution increased in patients with MI in the LAD compared to controls. These findings are as expected from the localization of LAD-MI in the anterior and septal walls (Fig. 2). Thus, this study has demonstrated how a chronic MI can affect novel parameters of LV pumping to different degrees depending on what coronary artery was occluded.

Our findings of preserved longitudinal contribution to LVSV are in line with the results of a previous study in patients with dilated cardiomyopathy [11]. In both patients with dilated cardiomyopathy and patients with MI, the AVPD was decreased but the dilated LV with a larger short-axis epicardial area resulted in a longitudinal contribution to LVSV that was similar to healthy controls. In contrast, patients with pulmonary regurgitation have decreased longitudinal and increased lateral contributions to SV in both the left and right ventricles [13]. The septal movement in patients with pulmonary regurgitation is towards the right ventricle resulting in a negative contribution to LVSV. The decrease in longitudinal contribution to RVSV was also seen in catheter-induced PR in an animal study and was reversible after percutaneous valve replacement [19]. Recently, a study on patients with pulmonary hypertension, found decreased longitudinal contribution and increased lateral contribution to LVSV similar to pulmonary regurgitation [17]. The preserved longitudinal contribution in patients with dilated cardiomyopathy or MI where the LV is primarily affected and dilated thus differs from patients where the RV is dilated because of pulmonary regurgitation and pulmonary hypertension. Further mechanistic studies are needed to explain the pathophysiology behind these differences.

We did not find any relationship between MI size and regional contributions to LVSV. This may be due to a limited patient population and larger studies may be needed to shed further light on a possible relationship. Larger sized myocardial infarction results in worse remodeling and reduced ejection fraction at follow up, and because of the larger myocardium at risk in anterior LAD-MI these are related to worse remodeling [20]. This was also evident in our findings of larger infarct size, lower AVPD and larger LV volumes in the LAD-MI compared to RCA-MI.

The reason for using the epicardial area of the LV to calculate the AVPD contribution to SV has been previously described and validated [10, 11]. Similar to any engine, when calculating the volume ejected due to a piston movement the entire area of the piston needs to be taken into account. The myocardial wall is incompressible and does not change volume during pumping, apart from the negligible small variations of volume caused by coronary blood flow, SV calculations are identical from endocardial or epicardial delineations. The re-arrangement of the myocardium during systole to a thicker wall is both caused by the longitudinal shortening resulting in thicker wall and the radial thickening per se of the myocardium. Furthermore, longitudinal pumping causes the majority of the thickening of the LV muscle and this thickening does not reflect LV radial pumping [18].

**Fig. 6:**
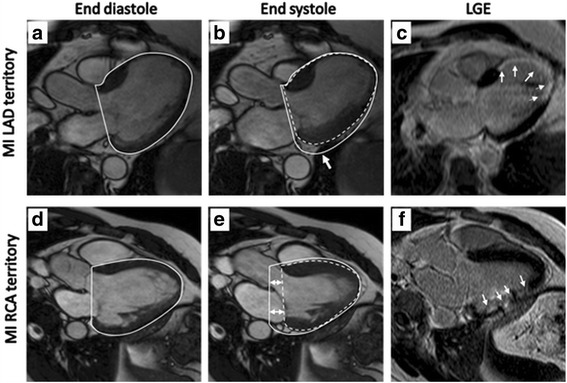
Illustration of differences in longitudinal, septal and lateral contributions to left ventricular pumping in a patient with LAD-MI (*top row*) and RCA-MI (*bottom row*). Cine images in the left-ventricular outflow tract view are shown in end diastole (left column) and end systole (*middle column*) and the corresponding late gadolinium enhancement (LGE) images (right column, **c** and **f**) show the extent of the infarcted area (*arrows*). The *solid white* outline of the LV in end diastole (**a** and **d**) is superimposed on the images in end systole (**b** and **e**) where the ventricles in end systole have been outlined with a dashed line. The AV-plane displacement (AVPD) is the difference of the *horizontal white line* at base of LV in end-diastole and end-systole and marked by *double arrow* at the base of ventricle in **e**. Longitudinal pumping is the difference in the basal contours at end diastole and end systole. Radial pumping is caused by the displacement of the epicardial border from end diastole and end systole. Note in **b** that the lateral component of radial pumping is increased (arrow at lateral wall in **b**) and compensates for the decreased longitudinal and septal contributions to LV pumping. As a contrast, the RCA-MI patient shown in the lower panels has a normal AVPD despite the MI. This may be compensatory to the decreased lateral contribution to LVSV in this patient due to the MI

**Fig. 7:**
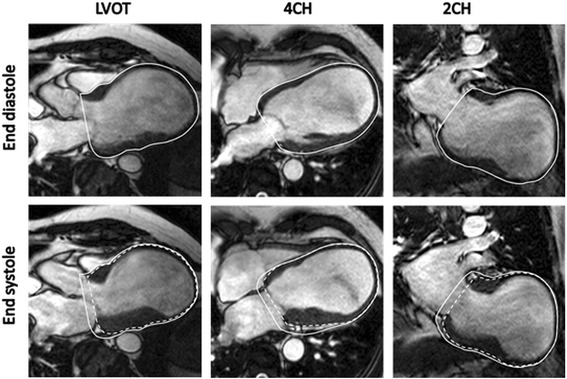
An example of a patient who has developed a very large apical aneurysm secondary to a myocardial infarction within the LAD territory. The *upper row* is in end diastole while the *lower row* is in end systole. In this case most of the stroke volume is generated by longitudinal AVPD (≈ 90%) of the viable myocardium at the base of the LV

The mean longitudinal contribution to LVSV on a group level did not differ from controls but the study of individual patients show differences in how LV remodeling after an MI affect cardiac pumping. Fig. 6 illustrates how the regional contributions to LV pumping can differ between patients. In the LAD-MI patient a large anteroseptal-apical infarct is seen in the LGE images and the septal epicardial contour does not shift during systole, resulting in a zero septal contribution to SV. This patient has a severely decreased AVPD and thus a smaller longitudinal contribution compared to both controls and the average of LAD-MI patients. To compensate for this decrease, there is an increased movement in systole of the lateral epicardial contour, resulting in a high proportion of lateral contribution to radial pumping. In contrast, the patient with an MI in the RCA territory has almost zero movement of the lateral epicardial contour, compared to the LAD-MI patient, i.e. decreased lateral contribution to SV due to the infarct in the lateral and inferior wall. This is compensated by a normal AVPD resulting in an increased longitudinal contribution to SV as the epicardial area in this patient is increased. These cases can be contrasted with a patient shown in Fig. 7 with a large LAD-MI and an apical aneurysm had 90% longitudinal contribution (Fig. 7). This patient had normal exercise capacity and few symptoms within one year after infarction. Thus, the increased piston-pumping of the AV-plane in this patient can produce a near normal aerobic capacity during stress. Thus, patients with LAD-infarctions and apical aneurysms can have both decreased and increased longitudinal function and the prognostic and functional importance of this finding remain to be elucidated.

In patients with MI a decreased AVPD is associated with worse prognosis [7] and recently Rangarajan et al. showed that a reduced lateral AVPD on CMR was an independent predictor of major adverse cardiovascular events [9]. Previous studies of measurements of left ventricular AVPD or mitral annular plane systolic excursion (MAPSE) by echocardiography have been used clinically to detect decreased ventricular function and shown to hold prognostic information in stable coronary artery disease [21]. However, it is not known if regional contributions to LVSV hold any prognostic information. Studies including larger patient numbers and prognostic information on e.g. exercise capacity, morbidity and mortality as well as potential additional diagnostic value from the AVPD, septal and lateral contributions to LVSV, would be of interest.

Automatic software for AVPD tracking exists and can be used to minimize user dependency of the AVPD measurements [22–24]. In addition, this provides additional information of the AVPD over the entire cardiac cycle. Examples of patient groups where AVPD is especially important are LV hypertrophy and patients with aortic stenosis where an increased radial movement compensates for the decreased longitudinal function [25]. Further studies to determine if the longitudinal/radial contribution to LVSV is affected in these patient groups are therefore warranted.

### Limitations

In this study, only patients with chronic MI were included. Thus, the results cannot be directly transferred to acute STEMI or non-STEMI. Further studies are needed to show if there is an effect on the different contributions to LVSV in acute MI and non-STEMI patients. Detailed coronary angiography data was not available in all patients, due to the retrospective nature of the study. Thus, we could not assess the procedural features in cases of percutaneous coronary interventions and the potential impact of the amount of vessel disease. Neither was time from pain onset to reperfusion during the STEMI available in this study and potential impact of remote ischemic preconditioning cannot be assessed.

The patient number in this study is limited and larger patient numbers may be needed to study the relationship between contribution of the different components of LV pumping to LVSV and infarct size. Of note, there was a numerically lower longitudinal AVPD contribution to SV in patients compared to controls, however this did not reach statistical significance. Further studies will show if this difference is statistically significant in larger patient materials. We did not include the effect of MI on RV function that may be seen in RCA-MI when part of the RV diaphragm wall sometimes is infarcted. Furthermore, in cases where the septum bulges to the RV during systole due to LV infarction, this movement will contribute to RV pumping. The effects of MI on the longitudinal, septal and lateral RV function may therefore be of interest for future studies. Finally, in this study we did not discriminate the contribution of the anterior, lateral and inferior wall of the LV in this study but combined them to one parameter called lateral contribution, which was compared with its septal counterpart. This was done to decrease the risk of type I errors due to a high number of comparisons in each subject.

## Conclusion

Chronic MI in the LAD and RCA vessel territory does not change the internal relationship between the contributions to overall LV pumping of longitudinal (≈60%) as compared to radial (≈40%) pumping. Although the overall contribution to SV of radial pumping did not change, MI within the LAD vessel territory caused a decrease in the septal component of radial pumping which seems to be compensated by an increase in its lateral component.

## Abbreviations

AVPD: Atrioventricular plane displacement
CMR: Cardiovascular magnetic resonance
EDV: End-diastolic volume
ESV: End-systolic volume
IS: Infarct size
LAD: Left anterior descending coronary artery
LGE: Late gadolinium enhancement
LV: Left ventricular
LVEF: Left ventricular ejection fraction
MAPSE: Mitral annular plane systolic excursion
MI: Myocardial infarction
PR: Pulmonary regurgitation
RCA: Right coronary artery
SD: Standard deviation
SE: Standard error of the mean
STEMI: ST-elevation myocardial infarction
SV: Stroke volume
